# Reflexive Practice as an Approach to Improve Healthcare Delivery for Indigenous Peoples: A Systematic Critical Synthesis and Exploration of the Cultural Safety Education Literature

**DOI:** 10.3390/ijerph19116691

**Published:** 2022-05-30

**Authors:** Jessica Dawson, Keera Laccos-Barrett, Courtney Hammond, Alice Rumbold

**Affiliations:** 1 School of Psychology, University of Adelaide, Adelaide, SA 5000, Australia; 2 Poche SA+NT, Flinders University, Bedford Park, SA 5042, Australia; 3 College of Nursing & Health Sciences, Flinders University, Bedford Park, SA 5042, Australia; keera.laccosbarrett@flinders.edu.au; 4 School of Public Health, University of Adelaide, Adelaide, SA 5000, Australia; courtney.hammond@sahmri.com; 5 Wardliparingga Aboriginal Health Equity Unit, SAHMRI, Adelaide, SA 5000, Australia; 6 Women and Kids Theme, SAHMRI, Adelaide, SA 5000, Australia; alice.rumbold@sahmri.com; 7 Adelaide Medical School, The University of Adelaide, Adelaide, SA 5000, Australia

**Keywords:** Indigenous, education, cultural safety, reflexivity, curriculum, pedagogy, assessment

## Abstract

Cultural safety is increasingly being taught in tertiary programmes of study for health professionals. Reflexivity is a key skill required to engage in culturally safe practice, however, there is currently limited literature examining how reflexivity is taught or assessed within cultural safety curricula. A systematic review of the literature up until November 2021 was conducted, examining educational interventions which aimed to produce culturally safe learners. Studies were limited to those with a focus on Indigenous health and delivered in Australia, Aotearoa New Zealand, Canada, and the United States. A total of 46 documents describing 43 different educational interventions were identified. We found that definitions and conceptualisations of reflexivity varied considerably, resulting in a lack of conceptual clarity. Reflexive catalysts were the primary pedagogical approaches used, where objects, people, or Indigenous pedagogies provided a counterpoint to learners’ knowledges and experiences. Information regarding assessment methods was limited but indicates that the focus of existing programmes has been on changes in learner knowledge and attitudes rather than the ability to engage in reflexivity. The results demonstrate a need for greater conceptual clarity regarding reflexivity as it relates to cultural safety, and to develop methods of assessment that focus on process rather than outcomes.

## 1. Introduction

The beliefs, attitudes, and biases that healthcare professionals hold can substantially influence the way they interact with and provide care to people. This is particularly relevant to the health and well-being of Aboriginal and Torres Strait Islander Australians, where healthcare professional attitudes are seen as a major factor in whether care is considered safe, adequate, and acceptable [1,2,3]. Dismissive, rude, and outright racist behaviours have been reported by Aboriginal and Torres Strait Islander people in their encounters with non-Indigenous healthcare professionals. These experiences can result in inadequate or negligent care provision and deter recipients of care from further engaging with a healthcare system that is perceived as culturally unsafe [4,5,6]. In turn, this can have significant flow-on effects for healthcare access and outcomes and contributes to the health inequities experienced by Aboriginal and Torres Strait Islander people. Parallels can be seen in Aotearoa New Zealand, Canada, and the United States, where racial discrimination and culturally unsafe care contribute to inequities in healthcare access and outcomes for Indigenous peoples [7,8,9]. 

Cultural safety is recognised as an approach to healthcare with the potential to improve the experience of care for Indigenous peoples in Australia, Aotearoa New Zealand, Canada, and the United States. The concept of cultural safety was originally developed in Aotearoa New Zealand by Māori nurses and midwives, to address the racism experienced by Māori patients being cared for by a largely non-Indigenous health workforce [10,11]. Cultural safety in healthcare delivery recognises the centrality of culture to health and well-being and seeks to ensure that healthcare is respectful and non-discriminatory. To provide culturally safe care, healthcare professionals need to engage in critical reflexivity, whereby they examine their own cultural identity, positioning and power, the values, attitudes, and biases they carry, and the potential consequences of these for the people they provide care to [12,13,14,15].

The importance of cultural safety as an approach to healthcare provision is now well established, with a substantial body of literature exploring the application of cultural safety across a range of health professions, including nursing and midwifery [16,17,18,19,20,21,22,23], medicine [24,25,26,27,28], psychology [29], physiotherapy [30], occupational therapy [31,32], and nutrition [33]. As such it is increasingly being included in health sciences curricula at the tertiary level in Australia, Aotearoa New Zealand, Canada, and the United States [34] and developed as short continuing professional education courses (for example, [35,36,37]). While there is a growing body of literature exploring best practice approaches to the development and delivery of cultural safety education, this predominantly focuses on overall curriculum structure and content (for example [20,31,38]). Currently, however, there is little guidance on the most appropriate pedagogical approaches to teach students how to be reflexive, or on how educators should assess student learning outcomes concerning reflexivity. Given the centrality of reflexivity to culturally safe practice, we argue that there is a need to develop best practice approaches to the teaching and assessment of reflexivity within cultural safety education. 

### Reflexivity and Cultural Safety

The way reflexivity is defined, conceptualised, and operationalised varies both across and within fields and disciplines, depending on the purpose to which it is being put [39,40]. At its core, reflexivity involves an awareness and examination of the ontological and epistemological foundations that inform our existence and shape our thoughts and behaviours [39,41]. Notably, reflexivity is concerned with the self in relation to others; that is, how our ways of knowing, being, and doing shape our interpretation of and behaviour towards people [40,42]. Despite its centrality to culturally safe practice, reflexivity has not been specifically defined or conceptualised in this context. In her account of reflexive, culturally safe research, Wilson draws on the broader research literature to define reflexivity as a tool “to gain greater understanding of the self/other positionalities and the experience of research” [42] (p. 219). How this might be adapted into healthcare contexts and included in cultural safety education is yet to be determined.

In the context of cultural safety, reflexivity serves several purposes, and each presents challenges for educators and students alike. Perhaps the most cited purpose of reflexivity is the examination of own cultural worldviews and values and how these might influence delivery of care to people from different cultural backgrounds [43,44]. Notably, there is an acknowledgement that ‘culture’ is a complex intersection of factors such as race, ethnicity, gender, age, socioeconomic status, disability status, geographical location, and sexual orientation, among others [34,43,44]. Arguably, this complexity presents students with a significant challenge of understanding their own intersectional nature. Given the tendency of cultural safety education to focus on Indigenous health, there is a risk that this type of curricula reinforces a false dichotomy of Indigenous and non-Indigenous identity, resulting in a curriculum more akin to cultural competency training [13,45]. It also risks diminishing the complexity of Indigenous identity, which encompasses more than ethnicity on its own [46,47]. Further, as Lumsden [39] (p. 3) notes, there is an inherent risk that reflexivity can easily become entangled in notions of individual identity, “while failing to recognize the wider disciplinary, institutional, and political context(s) in which reflexivity…takes place, and in which knowledge is constructed, situated, and (re)negotiated”. In cultural safety education, and specifically in the context of Indigenous health, considerations of these broader contexts and the sites and methods of knowledge (re)production are essential.

The development of reflexive skills and the exploration of own cultural identity most often occurs in conjunction with learning about the social determinants of Indigenous health. In colonised countries like Australia, Aotearoa New Zealand, Canada, and the United States, this includes developing an understanding of historical and ongoing processes of colonisation, and the resulting interpersonal and institutional racism, whiteness, and power differentials. Students also need to develop an understanding of how these factors intersect with other social determinants such as education, employment, housing, and food security to produce the health inequities that Indigenous people experience [13,43,48]. In part, this learning is intended to help students understand that health is the product of social, economic, political, and historical forces [44]. It is also an opportunity for students to become aware of and challenge their own internalised stereotypes, assumptions, and biases through exposure to new learning. Yet this can be a challenging process for students, one which has been consistently shown to produce feelings of discomfort for students [48,49,50]. These feelings of discomfort can range from disengagement in class to outright hostility towards content, learning, and educators. This discomfort, if carefully managed, can produce transformative learning experiences for students. Conversely, poorly managed discomfort may serve to reinforce negative attitudes towards learning, content, and Indigenous people themselves [48,49,50].

Additionally, students are expected to engage in this complex, deeply personal, and potentially uncomfortable learning process in the context of a tertiary educational institution. While educators might strive to provide genuinely transformative learning experiences, the reality for students is that they need to pass their studies. Faced with the task of engaging reflexively, there is a risk that students will simply provide the responses they think educators want. This has been acknowledged as a potential issue in the literature [39,49,51,52,53] but has not been explored in-depth regarding whether and to what extent this occurs within cultural safety education. 

While there is a growing body of literature on cultural safety education, at present, there is little guidance on the best approach to teach or assess reflexivity in the context of cultural safety. Currently, most cultural safety education literature falls into three broad categories: qualitative explorations of student learning experiences [18,22,30,54,55,56,57,58], evaluations of student learning outcomes [17,19,23,26,29,32,33,50,59,60,61,62,63,64,65,66], and descriptions of curricula development and delivery [16,20,21,25,28,31,38,67,68,69,70,71,72,73]. In evaluations of curricula and learning outcomes, student reflective journals are a common source of data. However, these articles tend to focus on whether and how the curricula have produced transformative learning, with little information provided on the specific pedagogical approaches used to teach students reflexivity, nor how assessments are structured to capture the reflexive process. 

The dearth of literature on teaching and assessment of reflexivity may be reflective of the diversity of definitions, conceptualisations, and ways of operationalising it as a concept. Lumsden [39] warns against using standardised instructions for learning how to engage in reflexivity, as this implies a ‘correct’ and ‘incorrect’ way of going about the process, and risks reducing it to a checklist approach. In the absence of such instruction, how do educators help students develop the skill of reflexivity? Further, in the tertiary education system, where educators are required to assess the learning outcomes of students in a standardised manner, how do they assess whether students have effectively demonstrated these skills? The current research aims to address these questions through a systematic synthesis and exploration of the Indigenous cultural safety literature, with a specific focus on reflexivity, and aims to address the following questions:

1. How is reflexivity conceptualised within cultural safety educational interventions?
2. Where and how is reflexivity included as part of learning outcomes in educational interventions?
3. What types of pedagogical approaches are used in cultural safety educational interventions to help students develop reflexive skills?
4. How is the development of reflexivity as a skill assessed?

## 2. Materials and Methods

### 2.1. Data Sources and Search Strategies

An initial systematic search of the following databases was conducted: CINAHL, PubMed, Scopus, Informit, PsycINFO, and Embase. Consultation with a research librarian determined that, due to the specificity of the search parameters and the limitations this placed on using indexing terms, a simplified set of search terms was most appropriate. Therefore, all searches across the databases used the following search terms: “cultural safety” and “culturally safe”. Where the databases provided the option, searches were limited geographically to Australia, Aotearoa New Zealand, Canada, and the United States of America, and to articles in English. 

Additionally, a targeted internet search was also conducted, specifically to capture data produced by Indigenous and other non-government organisations (NGOs) as well as any available information on CPE. These data sources represent an important but often overlooked source of information not captured in other literature reviews, as they are often direct accounts of the educational process and often centre Indigenous experiences, knowledges, and aspirations.

### 2.2. Eligibility Criteria

Data sources were included if they described an educational programme or intervention that fitted the following inclusion criteria: (1) aimed to develop culturally safe learners; (2) was delivered as either part of an undergraduate or postgraduate degree or as continuing professional education (CPE); (3) had a focus on Indigenous health outcomes; and (4) was delivered in Australia, Aotearoa New Zealand, Canada, or the United States of America. The latter criteria were employed as these countries share similar historical and ongoing colonial processes, with resulting similarities in health inequities experienced by Indigenous peoples. The inclusion criteria were designed to allow for the inclusion of all cultural safety education literature and the identification of variations in how and where reflexivity was included or excluded.

Data sources were excluded if they were published in a language other than English, described an educational approach other than cultural safety, or a full-text article was not available.

### 2.3. Article Review

The initial search of the databases returned a total of 2860 results, which were exported into Endnote [74]. Duplicates were removed (*n* = 1125), leaving 1735 results. Initial screening of titles was carried out by the lead author (JD), and identified a further 852 for removal, due to either irrelevance (for example, most of those excluded discussed a “culture of safety” rather than cultural safety), or duplicates missed by the Endnote sorting function. 

Titles and abstracts of the remaining articles (*n* = 883) were reviewed by JD and CH according to the inclusion/exclusion criteria; 738 articles were excluded, leaving 145 articles for full-text review. Full-text review resulted in the identification of another two articles, bringing the total number of articles reviewed to 147. JD conducted all full-text reviews, with a 10% cross-check provided by CH. Where agreement could not be reached on an article, it was discussed with AR until a decision could be made. An additional 29 articles were identified in the grey literature. An updated search conducted in 2021 identified an additional 17 articles for inclusion.

It should be noted that the targeted internet search identified a wide range of cultural safety training modules available via organisational websites, such as Australian Indigenous Doctors’ Association (Australia) and San’yas (Canada). Most of these organisational websites contained publicly available information about the expected learning outcomes of the training module but were excluded from analysis due to insufficient information on other aspects of learning. 

### 2.4. Data Extraction

Data in the current research are descriptive and primarily sourced from the introduction and background sections of articles where information about the educational intervention is provided as a preface to evaluation or measurement of student learning and outcomes. Data were analysed using a two-stage thematic analysis process. In the first stage, data were coded under four major themes drawn from the research questions: definition and conceptualisation of reflexivity; where and how reflexivity is included in learning outcomes; pedagogical approaches used to develop reflexivity; and assessment methods used to measure reflexivity development. The second stage of analysis used inductive thematic analysis, where learning is generated from the data itself rather than guided by existing theoretical frameworks [75]. Data in each of the major themes were iteratively analysed and coded according to the sub-themes that emerged. 

Additionally, discursive analysis [75] was used to provide a more nuanced understanding of how language practices shape the definition, conceptualisation, and practice of reflexivity within cultural safety education. Analysis of how reflexivity is defined drew on constructivist theory [76] to examine how language shapes our understanding of both the nature and purpose of reflexivity. Analysis of how reflexivity is conceptualised primarily drew on existing cultural safety and reflexivity literature (for example, [13,38,39,43]), and analysis of the pedagogical approaches drew on the object-based learning literature [77,78]. Throughout the analysis, we also drew on the work of Indigenous educators who operate at the cultural interface [79] and whose writings and approach to teaching are informed by Indigenous ways of knowing, being, and doing [53,55,80,81].

Data and analysis were managed using NVivo12 software [82].

## 3. Results

### 3.1. Summary of Educational Interventions

A total of 46 documents were analysed, describing 43 different educational interventions. The majority (*n* = 35) of documents described university-based educational interventions [16,18,21,22,23,24,25,26,27,28,29,30,31,32,33,38,54,55,56,57,58,59,60,62,63,64,66,68,69,70,73,80,81,83,84], with one set of documents describing a short vocational training course [85,86,87], and the remainder (*n* = 8) describing continuing professional education (CPE) courses for practicing health professionals [17,19,20,36,69,73,88,89]. Just over half of the documents analysed (*n* = 28) were from Australia [17,18,20,22,23,24,27,28,29,30,33,36,38,54,55,56,57,62,64,66,67,73,81,84,85,86,87,90], with the rest from Aotearoa New Zealand (*n* = 5) [25,61,71,72,89], and Canada (*n* = 13) [16,19,21,26,31,32,59,60,64,70,73,85,86]; there were no documents from the United States. Table 1 provides a summary of key characteristics of the documents included for analysis.

### 3.2. Definitions of Cultural Safety

While an exploration of cultural safety definitions was not a central aim of this study, it was notable that several definitions included did not contain any reference to reflexivity or similar processes, such as critical reflection or self-awareness. Six of the documents included for analysis made no reference to reflexivity or similar in either the definition of cultural safety or in the educational intervention [19,24,27,28,73,88]. One of these documents referred to practicing cultural safety skills learned in a previous topic via structured simulation workshops [28], but the cultural safety skills are not outlined so it is unclear whether this includes reflexivity. An additional eight documents included in the analysis did not include reflexivity in the definition or conceptualisation of cultural safety but did include reflexivity as part of the educational intervention described [23,32,33,56,57,64,67,68].

### 3.3. Definition of Reflexivity

Inductive thematic analysis of the data found a lack of consistency in the terminology used to name and describe the reflexive process within cultural safety educational interventions. Of the 46 documents analysed, 40 described some form of reflexive process. Ten documents specifically referred to a process of ‘reflexivity’, either as part of the cultural safety definition [17,18,25,26,29,30,31,84] or as part of the educational intervention [23,38]. The remaining 30 documents used variations of the following terms: (self) awareness [20,54,57,58,59,63,64,69,80,91]; (critical and/or self) reflection [21,22,32,33,36,55,56,57,58,63,64,66,67,68,73,80,81,83,84,85,86,87,91]; (self) examination [16,61,65,71,72,75]. None of the documents analysed used the term reflexivity as a standalone concept; the ten documents that used the term reflexivity did so interchangeably with the other terms listed above. 

Where documents described the process of reflexivity, a variety of terms was used. The most common descriptors included ‘reflect on’ [21,22,23,27,30,31,32,33,36,56,64,66,67,68,73,80,90]; ‘examine’ [16,17,25,61,71,72]; ‘become aware of’ [17,18,36,63,68,75,88]; ‘explore’ [17,18,36,63,68,75,88]; ‘consider’ [29,56,67,68]; ‘understanding’ [36,58,87]; and ‘identify’ [21,64,67,72,89]. What is notable about these descriptors is that most—including all the most commonly used—describe a passive process of identification, observation, and awareness. In contrast, some descriptors contain a call to action, for example, the requirement to use this new knowledge and understanding of self to enact attitude change [23,36,56,71,72], and to contest and deconstruct previous understandings [29,73].

### 3.4. Conceptualisations of Reflexivity

Four sub-themes emerged relating to how the purpose and focus of reflexivity was conceptualised. These sub-themes included self-identity; held beliefs; relationality; and context, with each sub-theme encompassing a suite of factors that learners were expected to reflect on. These factors are outlined in Table 2, below, although it should be noted that conceptualisations of reflexivity varied considerably across the data and inclusion of a sub-theme did not guarantee inclusion of all factors. All documents included for analysis conceptualised reflexivity using at least one sub-theme, but usually two or more sub-themes were present. 

#### 3.4.1. Sub-Theme 1: Self-Identity

The sub-theme of self-identity was primarily concerned with students reflecting on their own identity, culture, worldviews, and values, and was seen in 30 of the documents analysed [16,17,18,20,21,22,23,26,28,29,30,31,32,33,36,57,58,59,60,62,63,64,66,67,69,70,71,73,83,87]. The primary purpose of reflecting on self-identity was broadly described as developing an understanding that identity, culture, worldviews, and values are not universal, exemplified in the following extracts:

*“Participants were therefore encouraged to…explore their own culture, values, and beliefs [and] acknowledge difference”*

[17] (p. 248)

*“[Cultural safety] requires registered nurses to reflect on their own cultural identity and practice in a way that affirms the culture of clients and co-workers”*

[21] (p. 3)

Further, learners were required to develop an understanding that their own self-identity shapes and influences understanding, attitudes, and behaviours, as demonstrated in the following extracts:

*“This includes understanding your own worldview and how your values and beliefs influence the way you perceive other people”*

[57] (p. 88)

*“[Students will] reflect on their own cultural background and their life experiences including the development of values and attitudes that have shaped their thinking and behaviours”*

[23] (p. 120)

#### 3.4.2. Sub-Theme 2: Held Beliefs

Thirty-five of the documents analysed described the sub-theme of held beliefs, where learners were required to identify and articulate their current knowledge, attitudes, biases, power, and privilege specifically in relation to Indigenous peoples [16,17,19,20,21,22,23,25,26,29,30,31,33,36,38,54,55,57,58,59,60,62,63,64,66,67,69,70,73,80,81,83,87,90,91]. In most of these educational interventions, learners were required to reflect on both self-identity and held beliefs, and these were conceptualised as related. However, eight of the educational interventions only included a requirement to identify, reflect on, and in some cases actively critique held beliefs [19,25,55,56,68,86,88,89]. 

A closer examination of the language used to describe the sub-theme of held beliefs found that the process and focus of reflexivity was often couched in neutral terms that glossed over the inherent racism underpinning beliefs and attitudes, as seen in the following extracts:

*“…highlighted the importance of health providers reflecting on and questioning their own **assumptions** about Aboriginal people that can impact on the care they provide”.*

[90] (p. 3, emphasis added)

*“…learners were encouraged to reflect on their own cultural values or **emotional responses** to diverse histories, cultures, worldviews, values, and contemporary events related to Indigenous people”*

[32] (p. e2, emphasis added)

Notably, across the educational interventions, there was minimal expectation that learners would reflect on their future or current professional culture, and the norms, beliefs, and values that would inform their practice. Three exceptions to this are Demers et al. [31], Kelly et al. [20], and Ramsden [70]. Demers et al., note that cultural safety “requires exploration of cultures and identities, on both a personal and professional level” [31] (p. 184). Similarly, Kelly et al., argue that culturally safe nurses are “aware of their own culture and that of the hospital” [20] (p. 110), and Ramsden states that nurses and midwives must become aware of “the cultural boundaries which surround [the] traditional nursing and midwifery role” [70] (p. 23).

#### 3.4.3. Sub-Theme 3: Relationality

Half of the educational interventions (*n* = 23) described the sub-theme of relationality [16,17,18,20,21,22,25,29,31,36,54,57,58,59,60,64,66,67,69,70,73,87,91]. In this sub-theme, learners were required to reflect on how self-identity and held beliefs impact on engagement with and care for others, and how this contributes to poor health and social outcomes, as exemplified by the following extracts:

*“…students reflected on their own place-based identity (i.e., who they were, where they came from) and recognized how their own personal biases were unintentionally but significantly brought into practice and how those biases influenced their work and social interactions”*

[31] (p. 187)

*“…notice our own cultural practices and individual behaviours and the impact these may have on Aboriginal and Torres Strait Islander people”*

[87] (p. 23)

#### 3.4.4. Sub-Theme 4: Context

The fourth sub-theme identified in the data was context and was included in 10 of the educational interventions analysed [16,22,23,29,31,55,59,60,67,75]. Context was described as a process of reflecting on how self-identity, held beliefs, and relationality have been shaped by historical, social, political, and economic forces. Like relationality, reflection on context takes the process of reflexivity beyond introspection and allows a more critical analysis of the self as socially located. In some cases, this was a passive analysis, as in the following extract:

*“Become aware of their own social conditioning, their (often privileged) status, and how their conditioning and status can affect their interactions with clients”*

[59] (p.179)

In contrast, other conceptualisations of reflexivity recognised the opportunity to actively challenge and deconstruct [29] this social conditioning, a process that Sjoberg and McDermott refer to as “disassembling planks of belief” [73] (p. 30).

All educational interventions included content on contextual factors such as colonisation, racism, and sociopolitical processes, and their impact on the health and wellbeing of Indigenous peoples. It is notable that while there is ubiquitous recognition of how colonialism and racism have impacted on Indigenous health and well-being, there is comparatively less recognition of how these factors have shaped non-Indigenous self-identity, held beliefs, and relationality.

**Figure 1:**
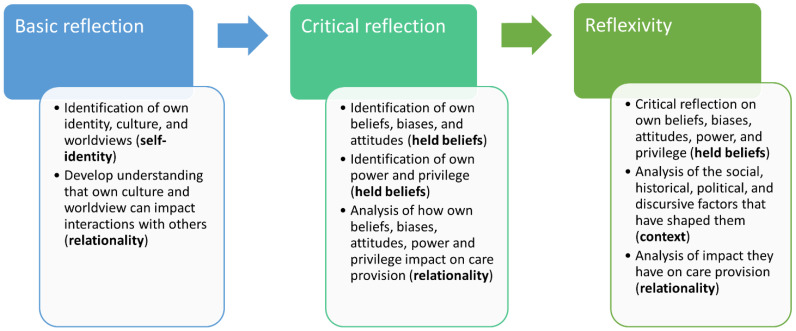
Spectrum of reflexive practice.

The varying conceptualisations of reflexivity can be seen as existing on a spectrum, ranging from basic reflection on self-identity at one end, to more critical reflection on self-beliefs and relationality in the middle, and reflexive analysis of the self as contextually situated at the other end. This can be seen in Figure 1, below:

### 3.5. Where and How Reflexivity Is Included as a Learning Outcome

None of the educational interventions included learning outcomes that explicitly required students to develop knowledge of or ability to engage in reflexivity. Instead, reflexivity was operationalised as a method for achieving other learning outcomes, such as developing awareness and knowledge of self-identity, held beliefs, relationality, and/or context. Of the documents included for analysis, only 15 explicitly stated the learning outcomes of the educational intervention, and of these, 10 included learning outcomes relating to reflexivity [17,29,54,55,56,59,64,70,85,91]. An example of this can be seen in the following extract, which outlines two of the learning aims of a one-day workshop for Australian postgraduate health sciences students:

*Specific aims of the day were to facilitate students in:*

*“Recognising and acknowledging their own views and frames of reference in relation to Indigenous Australians”;*
*“Critically reflecting on the impact of ongoing colonisation and its pervasive discourse on the health and well-being of Indigenous Australians”.*

[55] (p. 106)

In most of the other documents included for analysis (*n* = 31), expected learning outcomes could be inferred from the description of the educational intervention. For example, the extract below describes the intended learning outcomes of a community placement for master of physical therapy students in Canada:

*“Our goal was to study whether expanding the clinic beyond the classroom and into a Métis community would make the students more aware of their own identity and worldviews, how they may be different from those in that community, and how they shape their stereotypes and misperceptions of peoples from other cultures”.*

[58] (p. 147)

The conceptualisation of reflexivity as an approach to learning can be traced back to Ramsden’s early work on cultural safety education. The learning objectives outlined by Ramsden specifically require students to “examine their own reality and…attitudes”, “be open minded and flexible in their attitudes toward people”, and to become “self-aware” [70] (p. 22), learning requirements that are reflected throughout the cultural safety education literature. While there were a range of pedagogical approaches used to facilitate reflexivity, there is no mention of whether students were explicitly taught about the concept and purpose of reflexivity, or how to go about being reflexive. It may be that some of the facilitated activities included instruction on the concept and process, but this is not outlined in any of the literature included for analysis.

### 3.6. Pedagogical Approaches to Facilitate Reflexivity

The analysis revealed that reflexivity was facilitated through a range of pedagogical approaches, all of which involved some type of reflexive catalyst [78]. These catalysts were used as a counterpoint to the learner’s own lived experiences and worldviews, with the intention that exposure to difference would result in some level of reflexivity. Pedagogical approaches can be seen as grouped into three broad categories, depending on the type of catalyst used: objects, people, and Indigenous pedagogical practices. 

#### 3.6.1. Objects

Object catalysts were described in 27 of the educational interventions analysed, and included things like readings, case studies, stories, visual art, and films [17,20,23,24,29,30,31,32,33,36,38,56,59,62,63,64,66,67,68,69,71,73,84,85,87,90,91]. Reflexive engagement with object catalysts was most often depicted as small or large group discussions, where new knowledge and understanding was co-produced through the sharing of beliefs, experiences, and interpretations. Examples of this can be seen in the following extracts:

*“… the session includes a semi-formal lecture in a quieter space of the gallery … student literature reviews, gallery exploration, and a group reflective discussion”.*

[30] (p. 37)

*“The tutorial format was tightly structured and included the viewing of a vodcast (prepared specifically for the unit and featuring Aboriginal speakers), discussion of issues arising, case studies, and periodic presentations by students. … Guidelines [were] developed by students [to facilitate classroom discussions, to ensure] that consideration be given to experiences and background that may influence attitudes expressed”.*

[23] (pp. 115–116)

#### 3.6.2. People 

Twenty-six educational interventions analysed included people as a reflexive catalyst [16,17,18,19,21,22,24,25,26,27,28,31,36,38,54,55,56,57,58,59,60,62,63,69,80,81,83,84]. Immersive, community-based placements or field trips were the most common (*n* = 16), where predominantly non-Indigenous students visited (and sometimes stayed in) Indigenous communities or health services [16,18,22,24,25,26,27,31,54,57,58,59,60,63,81,83]. The educational interventions that took this approach shared similar theoretical underpinnings, where exposure to difference was described as an opportunity for reflection and growth. The extract below is exemplary of this theory:

*“Cultural immersion is an experiential approach to learning about culture and social situations. In medical education this type of approach is beginning to be recognized for its potential to raise consciousness among students; expose tacit inappropriate biases, including racism; help students learn about themselves and other cultures; and assist students in their preparation for work in culturally diverse settings”.*

[26] (p. 3)

In 13 of the educational interventions analysed, Indigenous educators were positioned as the reflexive catalysts, either as core teaching staff [17,36,56,57,68,71,90] or in ad hoc roles such as guest speakers [19,21,60,64] or patient simulation actors [28,63,86]. Indigenous educators were frequently described as providing learners with an opportunity to challenge their own stereotypes and beliefs about Indigenous peoples, as exemplified in the following extract:

*“Having the opportunity to be taught by and interact with an Indigenous academic is thought to have a major role in reducing stereotypes and negative attitudes about Indigenous Australians … Our objective was to present an Indigenous informed perspective filling in the gaps of knowledge that have resulted from silencing Indigenous peoples, their stories and experiences. We wanted to provide students with some positive and affirming images of the strength and resilience of Australian Indigenous peoples”.*

[55] (pp. 105–107)

Simulated patient scenarios involving Indigenous people as ‘patients’ were all described as an opportunity for learners to practice and refine communication skills and develop their cultural safety skills. This was seen as providing a safe, controlled environment for both learners and ‘patients’ where feedback could be provided to facilitate learner reflection [28,63,86].

#### 3.6.3. Indigenous Pedagogical Practices

Eighteen of the educational interventions analysed described the inclusion of Indigenous pedagogies as a method of facilitating learner reflexivity [16,17,18,19,27,30,32,38,54,56,60,64,68,71,73,81,83,84]. In some educational interventions this was an integral aspect of immersive community placements, where learners engaged in a variety of cultural and community events [16,18,27,55,61,85]. In other educational interventions, this was described as a process of engaging with Indigenous ways of knowing, being, and doing through activities such as talking (or yarning) circles [17,57,70,73], storytelling [19,30,32,68,90], and ‘Indigenised spaces’ [30,65,87]. In these educational interventions, Indigenous pedagogies were described as providing a counterpoint to deficit-based understandings that may be held by learners, or to legitimate Indigenous knowledges, as demonstrated in the following extracts:

*“field experiences can provide students with a first-hand account of the ‘ways of knowing, being, and doing’ …that is, communicating with and listening to Aboriginal Elders, practitioners and community members provides a deeper analysis of social work practice through assessing the cultural context, yarning and storying”*

[54] (p. 199)

*“A yarning circle approach was used to privilege First Peoples’ culture and voice. First Peoples have recognised yarning as a method of sharing stories, information and knowledge for generations”.*

[17] (pp. 247–248)

### 3.7. Assessment of Reflexivity

Of the 46 educational interventions analysed, only 22 provided information on the assessment of reflexivity, and in most instances only a brief description of the assessment task(s) was provided [18,22,23,24,26,27,29,33,38,54,55,56,58,59,63,66,69,73,81,84,85,91]. The most common forms of assessment were written reflections in the form of journals [23,24,29,57,60,87], essays [22,59,67,68,75], and portfolios [26,27,33]. Other forms of assessment included structured reflective questions [54,85], oral presentations [18], and arts-based reflection activities [59,63]. 

A common theme throughout assessments is the requirement for students to reflect on their learning, and how this applies to them personally and professionally, as demonstrated by the following extracts:

*“Students subsequently submitted a critical self-reflection exploring their personal learnings, including reflections on assumptions, discomfort and realisations”*.

[38] (p. 6)

*“The idea that students would be given a ‘real world’ experience, be required to reflect on what they had observed and what they had learnt, with supporting literature, was vital to identifying elements of changing attitudes and effectiveness of learning. Furthermore, students then had to plan how they would use their new knowledge in future practice contexts”.*

[54] (p. 201)

The above extracts demonstrate the two different ways that learners were required to reflect on their learning. In the first extract [38], the purpose of the assessment is described as assessing what students have learned about themselves, demonstrating an inward focus on self-identity and held beliefs. In the second extract [54], assessment is described as having a more applied focus; students must reflect on what they have learned and consider the implications for practice. 

Two documents included for analysis focused specifically on the assessment of reflexivity in their respective educational interventions, with descriptions of how these assessments connect to learning outcomes and are supported via learning activities. Sjoberg and McDermott [73] discuss what they call the ‘deconstruction exercise’, where students are required to critically examine and ‘deconstruct’ their chosen question rather than answer it directly. The aim is to expose the racialised assumptions and stereotypes that inform the question and how this links to broader social, historical, and political contexts. By externalising this critique, Sjoberg and McDermott argue that the deconstruction exercise provides learners with an “opportunity to reflect on the everyday language in which they may be immersed, to see behind the dominant Australian lexicon to the colonial, discursive position from which it has been constructed” [73] (p. 31). 

Power et al. [22] describe a reflective essay assignment in which students were required to complete three online reflections before, during, and after their 3-week placement. Students were provided with ‘trigger questions’ to prompt their online reflections, and these reflections formed the basis of their submitted reflective essay. The trigger questions step students through the reflective process, prompting them to think about their current knowledge and expectations (pre-placement), new learnings about themselves (mid-placement), and how this applies to future practice (post-placement). 

What is notable about the educational interventions described by Sjoberg and McDermott [73] and Power et al. [22] is that learners are supported to complete these assessments in several ways, including clear links made to topic content, scaffolded activities to support reflexive skill development, and prompting questions to guide the reflexive process. Several other educational interventions described similar approaches to support learner reflexivity, although only limited information was provided. For example, Chiodo et al. note that learners were required to keep a reflective journal in which they “reflect upon the topics covered in class and in the set reading material … to think about what the issues/concepts/theories…meant for them both in their personal and professional lives” [29] (p. 184). Here, assessment requirements are clearly tied to learning content and instructions regarding the focus of reflexivity; this was described in eight documents analysed [22,26,29,55,64,67,68,75].

## 4. Discussion

The current study analysed a total of 46 documents, which described 43 different educational interventions. Definitions and conceptualisations of reflexivity varied; in many definitions, reflexivity was conceptualised as a passive process of observation rather than an active process of analysis, critique, and change. Four sub-themes were identified: self-identity, held beliefs, relationality, and context, with conceptualisations of reflexivity drawing on varying combinations of these sub-themes. 

In all educational interventions analysed, reflexivity was considered an approach to learning rather than a learning outcome itself. Only 15 of the documents included for analysis specifically outlined syllabus learning outcomes, so it is possible that the other educational interventions include learning outcomes related to the development of reflexivity as a skill. Pedagogical approaches relied on three types of reflexive catalysts: objects, people, and Indigenous pedagogies. The use of reflexive catalysts was premised on the assumption that exposure to difference would engender understanding and respect for perspectives, beliefs, and experiences different to those of the learners. There was limited information available on the assessment of reflexivity, although most assessments focused on new knowledges and understandings gained through the educational intervention, including knowledge of self, and how these apply to students’ personal and professional lives.

It is noteworthy that 14 of the documents analysed included a definition of cultural safety that did not include reflexivity as a core aspect of culturally safe practice, with eight of those documents positioning reflexivity as additional to cultural safety, and six documents making no reference to reflexivity at all. Definitions that lack reference to reflexivity tend to align more with cultural competency models, which emphasises learning about other cultures and has been criticised as taking a more tick-box approach that risks essentialising culture and reinforcing stereotypes [13,45]. Cultural safety specifically moves away from this model of learning, with the emphasis placed on students learning about themselves and their own culture, and how power imbalances impact healthcare provision. Notably, there were several documents included in the analysis that used the terms ‘cultural safety’, ‘cultural awareness’, and ‘cultural competency’ interchangeably (for example, [21,23]) or conceptualised them as aspects or stages of the same process (for example, [17,36,86]). This potentially highlights a lack of understanding of core cultural safety concepts, and arguably, results in less effective teaching.

Variations in how cultural safety is conceptualised may provide some explanation for the variations in how reflexivity was defined and conceptualised. As noted by Lumsden [39], definitions of reflexivity differ according to context and purpose. The analysis showed that where the purpose of the educational intervention was to increase recognition and respect for diversity, reflexivity tended to be conceptualised as a process of understanding self-identity, held beliefs, and in some cases, relationality [16,17,18,19,20,22,23,25,26,28,29,30,31,32,33,36,38,54,55,57,58,59,60,62,63,64,66,67,69,70,73,80,81,83,87,90,91]. Where educational interventions were conceptualised as a way to address colonialism, privilege, and power imbalances, reflexivity was defined as a process of identifying and critiquing self-beliefs and the structural, institutional, and discursive factors that contribute to them [16,22,23,29,31,55,59,60,67,75]. 

As outlined in Figure 1, reflexivity could be conceptualised as existing on a spectrum. At one end, reflexivity was concerned with acknowledging and exploring self-identity and held beliefs, while at the other end, reflexivity was concerned with contextualising the self as socially located. Most of the documents analysed fell into the ‘basic’ or ‘critical’ reflection portions of the spectrum, with a greater focus on identification and understanding of self-identity and held beliefs, and to a lesser extent relationality. Expectations that students will identify and critique their self-identity and held beliefs would arguably be a contributing factor to student feelings of discomfort and resistance [48,49,50]. While discomfort is a necessary part of transformative learning, this discomfort needs to be carefully managed [49,50,54]. We would argue that a greater focus on the social, historical, political, and discursive forces which inform and shape students’ self-identity and held beliefs are an important part of the reflexive process. This would provide students the opportunity to understand that these are not immutable aspects of their own identity, but rather changeable aspects that have been shaped by problematic, inequitable, and racist systems [73]. If adequately managed, students may feel empowered to change problematic beliefs and attitudes while critiquing the systems that produced them.

All educational interventions analysed included information about the social determinants of health, so it is possible that learners were assisted to reflect on the connections between their own worldviews and broader contextual factors during learning. This is common throughout the cultural safety education literature, where learning about social determinants is positioned as a method for challenging and critiquing racialised beliefs, assumptions, and stereotypes that may negatively impact on care provision [20,33,42,43,56,57,68,71,72]. However, this was not evident from the available data, where discussion of the social determinants was explicitly described as developing an understanding of their impact on health outcomes for Indigenous peoples. Arguably this works to construct social processes as unidirectional, only impacting on Indigenous peoples’ health, without acknowledgement of how learners themselves are embedded within and shaped by these processes. This potentially limits the extent to which learners can engage in reflexivity, directing greater attention to self-identity and held beliefs and how they impact on behaviours and attitudes towards others. 

The pedagogical methods used to facilitate reflexivity are also worth greater examination. More than half (*n* = 26) of the documents analysed used people as the catalyst for reflexivity, where exposure to people with different cultures and life experiences provided learners with a counterpoint to their own culture, beliefs, assumptions, and stereotypes. Cultural immersion theory aligns with a pedagogy of discomfort [49], in that learners are taken out of their comfort zones and confronted with new knowledges and experiences that may challenge their preconceptions. What is notable here is that, while many of the immersion-based curricula were designed and delivered by Indigenous community members, there was little consideration given to the cultural safety of this experience for the community itself. In contrast, learner needs were paramount in discussions, with a range of strategies employed to manage student discomfort and create culturally safe learning experiences. For example, Gray et al. [62] describe an Indigenous health workshop for fourth-year allied health students, in which students interviewed local Aboriginal Elders and other community members to develop culturally safe communication skills. Gray et al., note that “this process provided a ‘safe space’ for students to interact with an Indigenous Australian person” [62] (p. 3). Arguably, however, there is at least as much risk for elders and other community members in the potential exposure to the racism, dismissive attitudes, and resistance to learning that often accompanies student feelings of discomfort [23,49,50]. Gray et al. indirectly acknowledge the potential for this to occur, noting that educators are taught how to de-escalate situations, and “post-workshop debrief sessions were held for teaching staff, to allow for the ‘venting’ of concerns” [62] (p. 3). 

In other immersive-based curricula, efforts were made to ensure that reciprocity was an underpinning principle, where communities received as much benefit as students did. This is exemplified by Hudson and Maar, who note that their placement experience was informed by a social accountability model, where “the obligation of medical schools is to direct education, research and service activities towards addressing priority health concerns in the community” [26] (p. 2). While laudable, it does not explicitly address the potential risks for the community members hosting students; there is an expectation that the risk to educators and other community members is worth the educational gains for students. Only two educational interventions explicitly addressed the issue of safety for Indigenous educators and community members [55,80], acknowledging the potential for Indigenous people to be exposed to racism. The culturally unsafe nature of the classroom for Indigenous educators is well recognised (see for example, [53,80,81]) yet there are currently limited strategies put in place to address this risk. Most educational interventions analysed had some level of Indigenous involvement in development and/or delivery (see Table 1), yet on its own this does not guarantee the safety of Indigenous educators or community members. There is a need for more research to develop strategies that minimise the risk for Indigenous people working in this space [92].

Finally, the lack of information on the assessment of reflexivity within cultural safety curricula highlights a significant gap in the literature. Reflexivity is a fundamental aspect of being culturally safe; presumably, then, it is important to determine whether learners have developed the necessary reflexive skills to become culturally safe. Yet assessment is often glossed over in curricula descriptions, with only brief summaries provided of what is being assessed. In all instances where information on assessments was provided, learners were expected to demonstrate reflection on learning and how this applied to them personally and professionally. There were no examples that required students to explicitly demonstrate reflexive skills; in other words, assessment was of content rather than process. Arguably there are issues with this approach; as noted previously, focusing on what students have learned about themselves is potentially problematic and could be a causative factor in student feelings of discomfort, disengagement, and resistance [50,75,80]. Additionally, the potential for students to game their reflections also calls into question the efficacy of these types of assessments. A possible solution is to shift the focus of assessment from content to process, where learners’ ability to demonstrate reflexivity is assessed, although currently there is very little research to indicate what this might look like (for example, [93]), and none within cultural safety education. Arguably then, there is a need for more research to determine how best to assess reflexive skills within cultural safety education without reducing it to either a checklist approach or a navel-gazing exercise [40]. 

While the results of this study are specifically concerned with reflexivity in the context of Indigenous cultural safety, cultural safety is increasingly being adopted in other discipline and population contexts. In particular, there is growing interest in how cultural safety might improve care provision and health outcomes for marginalised and disadvantaged populations, for example the LGBTIQA+ community [88,89], racial and cultural minority groups [94,95], and Indigenous populations globally [96]. In an increasingly globalized and multicultural society, the importance of cultural safety and the ability to engage reflexively is fundamental to the provision of equitable, non-discriminatory care.

### Limitations

A potential limitation of the current study is the type of literature that has been included. Most articles included in the analysis were evaluations of all or part of a cultural safety curriculum, with information on the learning outcomes, pedagogical approaches, and assessment options provided in the introduction or methodology sections. Articles were included where they provided sufficient information on at least three of the four key areas of analysis. The reliance on this type of data may explain the paucity of information on assessment approaches, as this was not a key feature of curriculum evaluations, where most of the focus was on changes in learner attitudes and knowledges, or learner experiences. However, the inclusion of this literature also means that a much broader picture of cultural safety education can be gleaned, compared to only including articles that focus on curriculum description.

Another possible limitation is that literature was only sourced from Australia, Aotearoa New Zealand, Canada, and the United States. It is possible that additional insights and perspectives might have been gained from other countries, broadening our current understanding of cultural safety curricula. Given the similarities in colonial history and Indigenous experiences of health and social inequity, it was felt that the cultural safety curricula literature would be comparable across these four countries, whereas this may not be the case for other countries.

## 5. Conclusions

While there is a substantial body of research exploring pedagogical approaches to teaching cultural safety in the context of Indigenous health, relatively little work has been done to determine best practice approaches to teaching and assessing reflexivity as a core cultural safety skill. Indeed, the above analysis demonstrates that even within the cultural safety education literature, there is substantial variation in whether and how reflexivity is included within definitions of cultural safety, and how reflexivity itself is conceptualised. This lack of conceptual clarity presents issues for educators when trying to develop cultural safety curricula and suggests that more work is required to develop a more cohesive model of reflexivity specifically aligned with the aims of cultural safety curricula and practice. Additionally, more thought must be given to the pedagogical and assessment approaches utilised within cultural safety education. A range of strategies were utilised during learning to manage student discomfort, yet almost no attention was given to how that discomfort might manifest in the context of assessments. Likewise, while there was a substantial focus on student safety within the educational interventions, relatively few educational interventions considered the cultural safety of Indigenous educators and community members involved in the development of delivery of these programmes. Further research is required to provide greater conceptual clarity, consistency in skills development, and safety of learners and educators alike.

## Figures and Tables

**Table 1:** Summary of key characteristics of the educational interventions included.

Reference Country	Learner Level and Field of Study	Site and Type of Educational Intervention	Duration. Delivery Method	Level of Indigenous Involvement
Arnold et al. (2008) [16] Canada	Undergraduate nursing students.	University. Reciprocal partnership, including community placements.	Not specified. Face-to-face.	Initiated, co-designed, and co-delivered.
Bernhardt et al. (2011) [59] Canada	Undergraduate speech-language pathology and audiology students.	University. Unit of study, including community placement.	8-month teaching period. 26 h in-class teaching time. 26 h out of class time. Face-to-face with some online components.	Advisory Group established. Co-designed and co-delivered.
Bolton and Andrews (2018) [30] Australia	Doctor of physiotherapy students.	University. Field trip within a unit of study.	Not specified. Face-to-face.	Co-designed and co-delivered.
Carriage et al. (2017) [24] Australia	Fifth-year medical students.	University. Rural and remote placements.	Half-day lecture followed by 5-week placement. Face-to-face.	Aboriginal Medical Service host organisations, including cultural mentoring.
Chiodo et al. (2014) [29] Australia	Undergraduate psychology students.	University. Unit of study.	6-week teaching period. 2 h weekly lectures. 1 h weekly tutorials. Face-to-face.	Includes a ‘diverse teaching group’ and involvement of ‘Indigenous guest speakers.’
Crampton et al. (2003) [25] Aotearoa New Zealand	Third-year medical students.	University. Cultural immersion placement.	1 week Face-to-face.	Consultation and collaboration with local community.
Delbridge et al. (2021) [38] Australia	Undergraduate and postgraduate health professions students.	University. 2 discipline specific PBL modules. 1 inter-professional simulation session.	PBL modules: Pre-workshop online learning, 1 h seminar, and 3 h workshop. Simulation: Pre-session online learning, length of session not specified. Face-to-face with some online components	Co-designed involving expert knowledge holders.
Demers et al. (2021) [31] Canada	Undergraduate occupational therapy students.	University. Fieldwork placement.	Pre-placement self-paced learning. 8-week placement. Face-to-face with some online components.	Partnership was initiated by a community-based Indigenous OT.
Dowell et al. (2001) [60] Aotearoa New Zealand	Third-year medical students.	University. Cultural immersion placement; part of a unit of study.	1 week Face-to-face.	Consultation and collaboration with local community.
Durey et al. (2017) [90] Australia	Health professionals (radiation oncology).	CPE. Workshop.	2 h workshop. Face-to-face.	Co-presentation.
Duthie et al. (2013) [54] Australia	Master of social work students.	University. Field experience; part of a unit of study.	1 day. Face-to-face.	Co-designed and co-delivered.
Fleming et al. (2017) [17] Australia	Midwifery academic educators.	CPE. Workshops and yarning circles.	2 half-day workshops and 5 yarning circles; held over a 12-week semester. Face-to-face.	Co-designed and co-delivered.
Gray et al. (2020) [62] Australia	Undergraduate allied health students.	University. Workshop.	1 day. Face-to-face.	Co-designed and co-delivered.
Hardcastle and Bradford (2007) [67] Australia	Nurses and other health professionals.	CPE. Online module.	6 self-paced learning modules. Online (web-based training programme).	Initiated, co-designed, and co-delivered.
Hart et al. (2015) [18] Australia	Undergraduate nursing students.	University. Pre-placement unit. Placement (urban, rural, and remote locations).	Pre-placement semester unit of study. Placement (5 weeks). Face-to-face.	Collaboration and consultation with Aboriginal Medical Services to set up placements.
Herzog (2017) [83] Canada	Fourth-year medical students.	University. Elective unit of study.	4 weeks. Face-to-face.	Development and delivery of learning.
Herzog et al. (2021) [68] Canada	Second-year medical students.	University. Class activity.	Not specified. Face-to-face	Not specified.
Hudson and Maar (2014) [26] Canada	First-year medical students.	University. Pre-placement preparation. Placement in community.	4 weeks (total) 2-week placement in Aboriginal community. 2-week follow-up on campus. Face-to-face with some online components.	Co-designed, co-delivered, and co-evaluation.
Hulko et al. (2021) [19] Canada	Health professionals (nurses working with dementia patients).	CPE. Module.	Self-paced, equivalent to 8–10 h completed over 8 weeks. Online and face-to-face components.	Co-designed and co-delivered.
Jackson et al. (2013) [55] Australia	Masters-level postgraduate health professions students.	University. Workshop within a compulsory subject.	1 day; 7 discrete sessions. Face-to-face.	Co-designed and co-delivered.
Jamieson et al. (2017) [32] Canada	First-year occupational therapy students.	University. Modules included in a first year OT course.	3 × 1 h modules. Face-to-face.	Co-designed and co-delivered.
Joyce (1996) [69] Aotearoa New Zealand	Undergraduate nursing students.	University. Scaffolded and integrated curriculum across undergraduate programme.	3-year curriculum. Approximately 252 h total across 3600 h of teaching. Face-to-face.	Co-delivery of teaching.
Kelly et al. (2016) [20] Australia	Renal health training for new and current nursing staff.	CPE. Workshop (pilot and evaluation).	Aim is to offer a 1-day workshop. Face-to-face.	Not specified.
Kickett et al. (2014) [56] Australia	First-year health sciences students.	University. Integrated curricula.	12-week semester. 2 h weekly tutorials. Offered in two formats: fully online; and face-to-face with some online components.	Co-coordination. Delivery of teaching.
Lucas et al. (2021) [57] Australia	Master of pharmacy students.	University. Immersive workshop.	8 h. Face-to-face.	Co-designed and co-delivered.
Maar et al. (2020) [80] Canada	Pre-clerkship medical students.	University. Simulated clinical scenarios.	15-min interview and 20-min debrief interview. Face-to-face.	Co-designed and co-delivered.
Mahara et al. (2011) [21] Canada	Baccalaureate nursing students.	University. Proposed curriculum. Scaffolded and integrated curriculum across the programme; includes a community placement.	4-year curriculum. Total amount of time not specified. Proposed activities would be face-to-face.	Conceptualisation, planning, and development.
McCartan et al. (2021) [33] Australia	First-year nutrition science students.	University. Integrated curriculum across first year.	Integrated across 4 semester-long first-year subjects. Face-to-face.	Co-designed.
Mills et al. (2022) [84] Australia	Undergraduate health sciences students.	University. Semester-long unit of study.	Four 3 h workshops across a 12-week unit. 1 face-to-face workshop; 3 online workshops (due to COVID-19).	Co-designed and co-delivered.
Min et al. (2020) [63] Canada	Third- and fourth-year pharmacy students.	University. One-semester unit of study; includes experiential learning activities.	3 h per week; 36 h total. Face-to-face.	Co-designed and co-delivered.
Nash et al. (2006) [64] Australia	Undergraduate nursing students.	University. Scaffolded and integrated curricula across the programme of study.	Seven units across the programme had content embedded; five were practical placements. Face-to-face with online components.	Consultation and collaboration in the development.
NSW Government Family and Community Services (2007) [85,86,87] Australia	Not specified.	Vocational training. Units of study within a Certificate III in Aged Care.	5-day workshop. Face-to-face.	Contributed to resource development. Required as assessor(s).
Oosman et al. (2019) [58] Canada	Master of physical therapy students.	University. Pre-placement orientation session. Placement in community.	Varied length, 2–4-week placements. 2 days per week in a health facility, 3 days per week in community. Face-to-face.	Design and delivery of community practicum.
Paul et al. (2019) [27] Australia	Medical students, first to fourth year.	University. Vertically and horizontally integrated curriculum.	Activities included in the curriculum vary between 1 h (smoking ceremony and welcome to country) and 8-weeks (rural GP and psychiatry rotation). Face-to-face.	Aboriginal health team responsible for coordination, development, implementation, and evaluation.
Power et al. (2020) [22] Australia	Third-year nursing students.	University. Elective clinical placement.	Not specified. Face-to-face.	Written and facilitated.
Ramsden (1992) [70] Aotearoa New Zealand	Undergraduate nursing and midwifery students.	University. Proposed curriculum framework.	Not specified, but curricula to be scaffolded and embedded throughout the programmes. Not specified.	Conceptualisation of framework.
Richardson et al. (2017) [71] Canada	Child and youth mental health workers.	CPE. Short course.	5-day training programme. Face-to-face.	Co-designed and co-delivered.
Royal Australian College of General Practitioners (2011) [36] Australia	Medical practitioners.	CPE. Framework for delivery; to be developed and delivered on a case-by-case basis by accredited trainers.	Minimum 6 h, up to 10 h of structured learning. Must also include preparatory activities. Mandatory 6 h face-to-face; can also include online components.	Planning, delivery, and evaluation of the programme.
Ryder et al. (2013) [28] Australia	Second- and third-year medical students.	University. Structured clinical simulations.	3 h session. Face-to-face.	Co-designed and co-delivered.
Sjoberg and McDermott (2016) [73] Australia	Health professions students (undergraduate and postgraduate).	University. Assessment included within a semester-long unit of study.	Not specified. Face-to-face.	Development.
Thackrah and Thomson (2013) [23] Australia	First-year midwifery students.	University. Semester-long unit of study.	12-week semester. 2 contact hours per week. Face-to-face.	Co-designed and co-delivered.
The Royal New Zealand College of General Practitioners (n.d.) [91] Aotearoa New Zealand	Practicing general practitioners.	CPE. Online training module.	Self-paced training module. Online.	Development and presentation.
Thorpe and Burgess (2012) [66] Australia	Undergraduate preservice teachers.	University. Semester-long unit of study.	12-week semester. Weekly contact time not specified. Face-to-face.	Co-designed and co-delivered.
West et al. (2021) [81] Australia	Final year undergraduate podiatry students.	University. Immersive clinical placement.	Minimum of four 1-day placements over the final year of study. Face-to-face	Clinic is staffed by Aboriginal health professionals.

**Table 2:** How the purpose and focus of reflexivity is conceptualised: sub-themes identified in the data.

Self-Identity	Held Beliefs	Relationality	Context
Identity Culture and ethnicity Worldview Values	Assumptions Biases and stereotypes Internalised racism Power and privilege	Impact of self-identity and held beliefs on relationships with others	Impact of context on self-identity, held beliefs, and relationality

## Data Availability

All data are publicly available as this is a review article. Data are summarised in Table 1 (Summary of key characteristics of the educational interventions included).
